# Residential wearable RSSI and accelerometer measurements with detailed location annotations

**DOI:** 10.1038/sdata.2018.168

**Published:** 2018-08-21

**Authors:** Dallan Byrne, Michal Kozlowski, Raul Santos-Rodriguez, Robert Piechocki, Ian Craddock

**Affiliations:** 1 SPHERE, Electronic Engineering Digital Health, University of Bristol, Office 1.31, Merchants Venturer Building, Bristol BS81UB, UK

**Keywords:** Electrical and electronic engineering, Computer science

## Abstract

This repository offers smart-home wearable accelerometer and Radio Signal Strength Indicator (RSSI) data acquired : 1) with low-cost hardware; 2) with high-resolution location annotations; 3) from four UK homes. The data are intended to evaluate RSSI-based indoor localisation methods with activity measurements provided from a user-worn wearable device. A wrist worn accelerometer records activity signatures which are relayed to a number of receiving Access Points (AP) placed throughout the building. Upon reception of a packet, each AP measures the RSSI of the received radio signal and timestamps the accelerometer measurements. Location labels are recorded automatically using a small camera which registers fiducial floor tags as the participant carries out their normal routines in a natural way. Approximately 14 h of annotated wearable measurements are provided. A scripted fingerprint measurement is provided along with several unscripted natural living recordings, where the participant carried out a number of daily household activities which are annotated, where possible, throughout. Codes are provided to access the data and to replicate the ground-truthing procedure.

## Background & Summary

Low-cost, networked, smart-home technologies can be used to alleviate the burden faced by national health services, freeing up valuable resources for patients requiring acute treatments. Localising patients within the home can provide unique insights into their behavioural patterns when undergoing physical or mental rehabilitation. Received Signal Strength (RSS) based localisation methods are ideally suited for low-cost smart-home systems deployed as an assistive health technology^1^. A patient wears a Radio-Frequency (RF) device which transmits salient information to a number of static receiving nodes placed around the home. Each static node (receiver) measures the RSS Indicator (RSSI) which represents the received RF power of an individual packet of wearable data as it propagates from the wearable RF device (transmitter). RSSI can be analytically related to distance under ideal Line-Of-Sight (LOS) conditions, however this relationship no-longer holds when physical obstacles or human-body shadowing distorts the RF propagation channel^2,3^.

A predictive localisation model may be used to determine the users location based on observing changes in this RSSI signature over time^1,4,5^. Evaluating a model which will work with users in a residential setting requires significant amounts of annotated measurements, capturing the ground-truth locations at a high resolution without significantly altering the individuals natural behavioural patterns. Several studies contain RSS Wi-Fi measurements that span multiple floors of University buildings^6–10^, while others focus on data gathered in single room^11,12^. Each study provides reference labelled locations. The location coordinates were obtained: manually from the participant using floor-plans in ref. 7; via a smart-phone application with a reference map^6,8,11^; using predictive proximity estimates from an algorithm^9,13^ or using a laser range finder, above head height, fitted to a scaffold with a laptop^10^. These labelling methods provide reasonable location approximations but are not well suited to a residential setting. Users often perform brief movements within their home, frequenting locations that coincide with activities such as washing oneself in a bathroom, cooking in a kitchen and relaxing on the sofa or in a bed. To record detailed location data, a user would be required to break frequently from their routine to provide course location labels, which is at odds with the purpose of studying their behaviour at home.

Popleteev *et al.*^10^ provided accurate high-resolution locations using laser methods for a dataset of ambient RF signal data (ambient FM, GSM and television signals) in a single residential apartment. These measurements were taken when the user was stationary as free-living or motion experiments cannot be annotated due to the significant weight and form-factor of the ground-truthing equipment. The SPHERE challenge activity recognition dataset provides room level-annotations in a single residential home^14^. Labels were provided by a third party who manually annotated a video sequence of the user. High resolution, sub-room level location labelling and participant pose extraction would incur a significant labour cost for the annotator and would thus limit the amount of experimental data that can be recorded.

**Figure 1:**
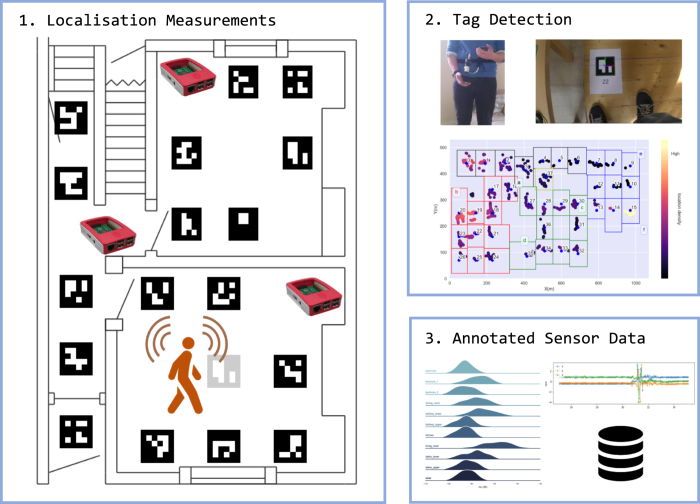
Block diagram describing the data collection, tag detection and labeling. 1. Tags are placed at specific locations around the residence. The user wears a transmitting wearable and Raspberry Pi receivers are placed throughout. 2. As the user traverses each tag, the downward camera, strapped to the torso, records frames containing the tags. The tags are processed using the provided scripts and the relative camera pose and position are obtained to provide annotated occupancy positions. 3. The recorded wearable accelerometer and RSSI measurements can now be annotated to a particular participant position within the house.

**Figure 2:**
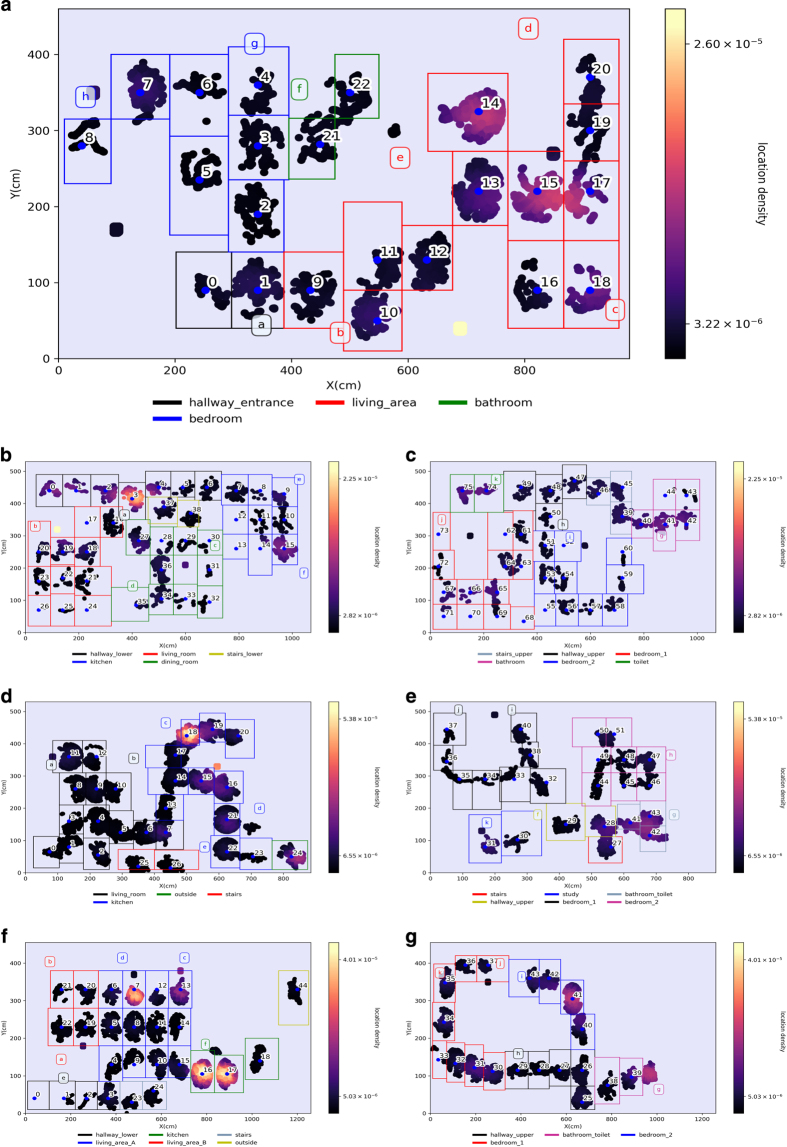
User occupancy Gaussian KDE heatmaps from each residence. Derived from all unscripted living experiments in houses (**a**) A; (**b**) B floor 1; (**c**) B floor 2; (**d**) C floor 1; (**e**) C floor 2; (**f**) D floor 1; (**g**) D floor 2. The blue dots highlight the tag centre coordinate on the floor while the access points are illustrated using alphabetic characters. The annotated locations associated with each received wearable packet, pertaining to accelerometer measurements and an RSSI signature, are collated from each free-living experiment for a particular home. The annotated locations are then filtered to 0.4 m from the tag centre and a two dimensional Gaussian KDE is applied where the bandwidth is estimated using the Scott's rule^21^.

This dataset registers high resolution annotated RSS and accelerometer data from scripted and natural experiments occurring at four residential homes. Ground truth location labels are provided by placing binary encoded floor tags at one metre spacings throughout the home. An automated system is presented using a downward facing camera to capture the encoded floor markings and provide the ground-truth location of the participant while minimising their influence on the annotation process. From the processed video, the user pose and the relative position of the chest-strapped camera can be obtained from each frame, increasing the resolution of the location labels beyond a square metre. A block diagram in Fig. 1 illustrates an example tag layout and highlights the annotation and labelling process. The contributions herein are:

- The measurements span four residential premises which, to the best of the authors knowledge, mark this RSS-based localisation dataset as the most comprehensive within the literature.
- All 14 h of measurements are annotated with high resolution (see Fig. 2) location labels, capturing the participant's movement and natural behaviour within the home.
- The data are accompanied with sample codes to peruse the measurements as well as scripts to replicate the annotation procedure.

Each dataset contains a typical fingerprint sequence where the participant stands at every floor tag in the residence, facing each of the the four cardinal directions. Aside from this, there are unscripted living experiments that span 8.5 h, where the participant was encouraged to carry out their normal routine of daily living. A number of tags were used to mark activity-centred locations such as the bathroom sink, kitchen area, sofa, beds, dining table chair and desk chair. Several three to five minute sequences are also presented where the participant was asked to continuously walk throughout the home. There were 11 receiving APs placed in three homes and eight within the one-bedroom apartment (Residence A). The resultant files can be examined using the example Python code provided. C++ codes are also provided to create and detect floor tags with recorded video data. A Python module is present which detects Bluetooth Low Energy (BLE) advertising packets on any AP running a linux-based operating system. These data can be used to benchmark indoor localisation methods which incorporate RSSI and/or accelerometer measurements from a wrist worn wearable device^5^.

## Methods

### Hardware

The participant wears a wearable transmitter on their wrist which contains a tri-axial accelerometer recording at 25Hz. The wearable component package is based around a Texas Instruments CC2650 system-on-chip SoC with a Cortex M3 processor. The ADXL362 accelerometer supports measurements of +/−4 g (9.81 ms^−2^). Five measurements are concatenated and transmitted via BLE radio at a rate of 5 Hz^15^. BLE advertising mode is used for transmission to ensure maximum coverage and extended range. Full details regarding the construction of the wearable are given in ref. 15. Commercial off-the-shelf alternatives with Bluetooth 4.0 and an embedded accelerometer are available and can be integrated as a wearable if configured to operate in BLE advertising mode^16^.

A Raspberry Pi (3 Model B) computer is used as a receiving AP. The BLE reception is handled by an on-board printed antenna and a combined Wi-Fi/Bluetooth BCM43438 chip. A Python script is used to configure the BlueZ driver and extract the salient accelerometer readings from each advertising packet. A single 10 AHr USB power bank was used to power a Raspberry Pi in select locations which did not contain an electrical outlet, i.e. bathrooms.

A Panasonic HX-A500E-K video camera with form factor (0.026 m×0.069 m×0.026 m) is used to capture the ground truth information. Video is captured at 25 FPS at a resolution of (1920×1080) and encoded using H264 at 15 Mbps.

### Ground Truthing

The camera is attached to the participant, facing towards the floor. For the experiments herein, the participants used a short plastic clamp to ensure the camera was approximately 0.1 m from the navel area of the torso, highlighted in the left side photograph in Fig. 1. As the participant moves around the environment, the camera captures video of floor tags placed throughout.

Numbered floor tags were used to obtain a reference location to label the participant's location. Each tag is a square binary image representing an encoded representation of an integer^17^. The square edges of the tag are used to infer the camera pose information and the encoded binary ensure that the detection process is robust in the face of poor lighting conditions, video distortion, angles and distances. A checkerboard of tags (provided) is used to calibrate the camera to counter the distortion of the lens. A 1024 element dictionary is generated^18^ and the fiducial marker tags are created using OpenCV^19^ with example code provided within the repository. The relative orientation and location of the camera, with respect to the floor tag, can be extracted from the video after the experiment is complete using the codes provided. The Panasonic HX-A500E-K camera was synchronised to the same NTP clock as the Raspberry Pi APs to maintain consistency with the timestamped wearable data. A digital display of the NTP clock is placed in front of the camera at the beginning and end of each video. These Coordinated Universal Time (UTC) timestamps are then synchronised to the camera clock time using the ELAN annotation software tool^20^.

### Residences

Four separate residences were considered for data acquisition in this study. The full description of each house is contained in the dataset including: a) floor and activity tag coordinates; b) AP coordinates; c) room names; d) floor tag adjacency list; e) tags and APs pertaining to each room; f) details of the five activity zones. Table 1 summarises the characteristics of each residence.

Only eight (out of 11 available) APs were deployed in residence A as the space was significantly smaller than the other houses. This coincided with a limited number of electrical sockets to power the APs. Fig. 2 illustrates the floor tag layout associated to each room within each home. The floorplan blueprints have been omitted on ethical grounds as the authors were not granted permission to use them. The kitchen, living and dining area of A (Fig. 2a) are within a single room. The third bedroom in C is used as a study with a desk as shown in Fig. 2e. The kitchen and dining area of C are a single room (Fig. 2d). The living and dining area of D are a single room, illustrated in Fig. 2f, but denoted as two in the dataset due to the significant area of both. The stairs of Residence B are split into two levels as the bathroom resides on a third tier between floor one and two, as shown in Fig. 2c. Back garden space at C and D allowed for a single tag to be placed outside, illustrated in Fig. 2d and f, respectively. Activity zone tags were placed at five separate locations in each home that were linked with particular activities, described in detail in Table 2.

### Experiments

Tables describing the experimental measurement data captured at residences A, B, C and D are given in Tables 3, 4, 5, 6, respectively. Each participant is labelled as User and their id lettering is the lowercase of the residence at which they resided, i.e. user a resided at House A, c at house C, d at D. There is no homeowner from residence B as this is an experimental residence owned by the University of Bristol. a and b are authors in the paper and c is known to the authors but does not work at the University. Each participant was fully informed and provided consent to participate in the study. The study falls under an approved UK Health Service Executive Health Research Authority Research Ethics Committee application, number 17/LO/0283.

The only consistent scripted experiment across all four dwellings are the four orientation fingerprint measurements where a participant spent a minimum of 20 seconds at each of four 90 degree orientations. Zero degrees corresponds to the users orientation when parallel with the tag as shown in the right side photograph in Fig. 1. The fingerprint is an example of a typical training sequence for RSSI localisation models^1^. Residence B, C and D contain a *fingerprint_rapid*, where the participant was asked to walk around, unscripted, visiting every floor tile in the house. B, C, and D, also contain training sequences for each of the five activity zones in *fingerprint_activity*. The authors did not have sufficient time for the collection of either *fingerprint_rapid* and *fingerprint_activity* in Residence A but suggest that *walking_rapid* and *walking* may be combined to emulate *fingerprint_rapid* for RSSI signature training.

In House A and B the users performed experiments of continuous walking throughout as highlighted in Tables 3 and 4. All other unscripted experiments are labelled as *living*. Here the user was encouraged to go about their normal routine. Activities were later annotated using the custom activity zone markers (highlighted in Table 2) and the videos were manually checked and annotated to ensure the correct activity time-span was recorded. Fig. 2 illustrates the participant location in each home using Gaussian KDE occupancy maps from all living experiments combined. The annotated locations associated with each received wearable packet, pertaining to accelerometer measurements and an RSSI signature, are collated from each free-living experiment for a particular home. The annotated locations are then filtered to 0.4 m from the tag centre and a two dimensional Gaussian KDE is applied where the bandwidth is estimated using the Scott's rule^21^.

### Code availability

Code resides in the “codes” sub-directory of the repository consisting of:

- a Python module with examples on how to load and examine the experiment data detailed in the paper;
- a Python module to scan for BLE advertising packets on a Raspberry Pi;
- C++ code to generate a floor tags, generate a camera calibration board, detect floor tags and extract pose information from the video.

## Data Records

There are four directories for each residence labelled house_<house_id> where <house_id> is either A, B, C or D. Each directory contains two sub-directories: one that contains the house and hardware metadata; and the second with a set of experiments listed in one of Tables 3, 4, 5, 6.

### Residence Metadata

Each home contains a list of .dat files which are written in csv format. A table of each file and description of it's contents are given in Table 7. The first column of each file can be treated as an index key with subsequent columns describing the values pertaining to that key.

### Experiment Directory

In each experiment directory, there are a number of csv.dat files describing the acquired RSSI and accelerometer recordings received by each AP. Table 8 outlines a list of files and a broad description of each.

The received accelerometer and RSSI data are provided in rx_wearable_data.dat. Each row represents a received wearable packet:

- timestamp : UTC timestamp when the packet was received by an AP;
- ap_id : the receiving AP index which corresponds to the keys in ap_mac_ref.dat;
- wearable_id : the index of the users wearable device which corresponds to the keys in wear_mac_ref.dat;
- rssi : the RSSI value in decibels (dB) assigned by each AP;
- acc<n>x,acc<n>y,acc<n>x,n∈{1, 2, …, 5}: five consecutive tri-axial accelerometer readings in units of g (9.8*ms*^−2^);
- batterylevel : the battery voltage level of the wearable device;
- seqno : the sequence of transmitted packets from the wearable.

Each packet received contains five sequential accelerometer measurements and the same packet is often received at multiple APs. The accelerometer measurements from rx_wearable_data.dat (acc<n>x,acc<n>y,acc<n>x, n∊{1, 2, …, 5}) are filtered to remove redundancy and rewritten into sequential order of measurement in accelerometer_filtered.dat. Each row describes:

- timestamp: UTC timestamp when the first accelerometer measurement arrived at an AP;
- acc_rx_packet: Sequence number of this accelerometer reading from the initial packet received (n∈{1,2,…,5});
- wearable_id: the index of the users wearable device;
- seqno: the sequence of transmitted packets from the wearable;
- acc1x,acc1y,acc1z : tri-axial accelerometer readings in units of g;
- acc1mag : magnitude of tri-axial accelerometer measurements.

tag_annotations.dat contain the annotated location of the participant throughout the experiment. Each row denotes:

- timestamp : UTC timestamp of the video annotation;
- timestamp_video : original video time in seconds;
- tag : floor tag number detected;
- tag_coord_x,tag_coord_y,tag_coord_z : coordinate (x,y,z) of the camera relative to the tag;
- orientation_wrt_tag : orientation of the camera (in degrees) relative to the tag.

All tags were placed along the same orientation at each residence parallel to the side walls, therefore the camera pose approximates the user pose within the residence. tag_coord_x,tag_coord_y,tag_coord_z can be offset directly with the tag coordinates listed in the house metadata file tag_coordinates.dat (Table 7). The code provided contains examples to load and combine the location labels from tag_annotations.dat with the wearable experiment data in rx_wearable_data.dat.

A supplementary annotation file activity_annotation_times.dat is presented to clarify the full time spent at a particular activity zone. On occasion, the participant would register in an activity zone tag once or at the beginning and end of their time at the activity zone. Therefore the full activity zone times were manually re-checked and annotated subsequent to data collection, using the ELAN software^20^. Each row of the file describes:

- timestamp_start : UTC timestamp when the activity began;
- timestamp_end : UTC timestamp when the activity finished;
- activity_tag : the tag corresponding to this activity which can be referenced with act_desc.dat in Table 7.

movement.dat contains labels of sequences of times where the user was stationary or walking continuously. Note that stationary does not necessarily mean standing still and can involve sitting and lying. Each row contains:

- timestamp: UTC timestamp of the annotation;
- motion_status: behavior description, can be either "S" for stationary, "W" for walking (moving) or "-" for undefined activities. The derivation of the motion_status labels is described in the next Section.

## Technical Validation

### RSS Measurements

**Figure 3:**
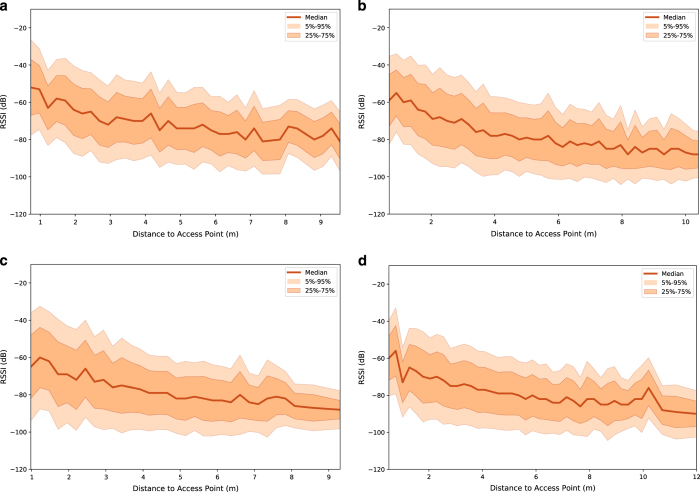
Relationship between RSSI and the distance from the AP for the fingerprint_floor experiment. Median RSSI values, 5–95% and 25–75% confidence intervals are included. Locations are taken within 0.3m from the floor tag centre coordinate. All four orientation angles are included. (**a**) House A (Pearson's (*ρ*) coefficient value=−0.445). (**b**) House B (*ρ*=−0.462). (**c**) House C (*ρ*=−0.406). (**d**) House D (*ρ*=−0.388).

The recorded RSSI measurements are compared directly to the participant distance from the AP where the median RSSI values, 5–95% and 25–75% confidence intervals are illustrated in Fig. 3. All APs are collated and measurements were included when the participant was less than 0.3 m from the floor tag centre coordinate during the fingerprint_floor experiment in each home. All four orientation angles are included. There is a negative coefficient from each of the Homes, with all magnitudes registering a Pearson's coefficient (*ρ*) below 0.5. While the median RSSI presents a course visual trend, the deviation is significant in all homes and the *ρ* values do not indicate significant linear correlation between the RSSI and the distance of the wearable device from the AP for these particular locations in a residential domicile.

**Figure 4:**
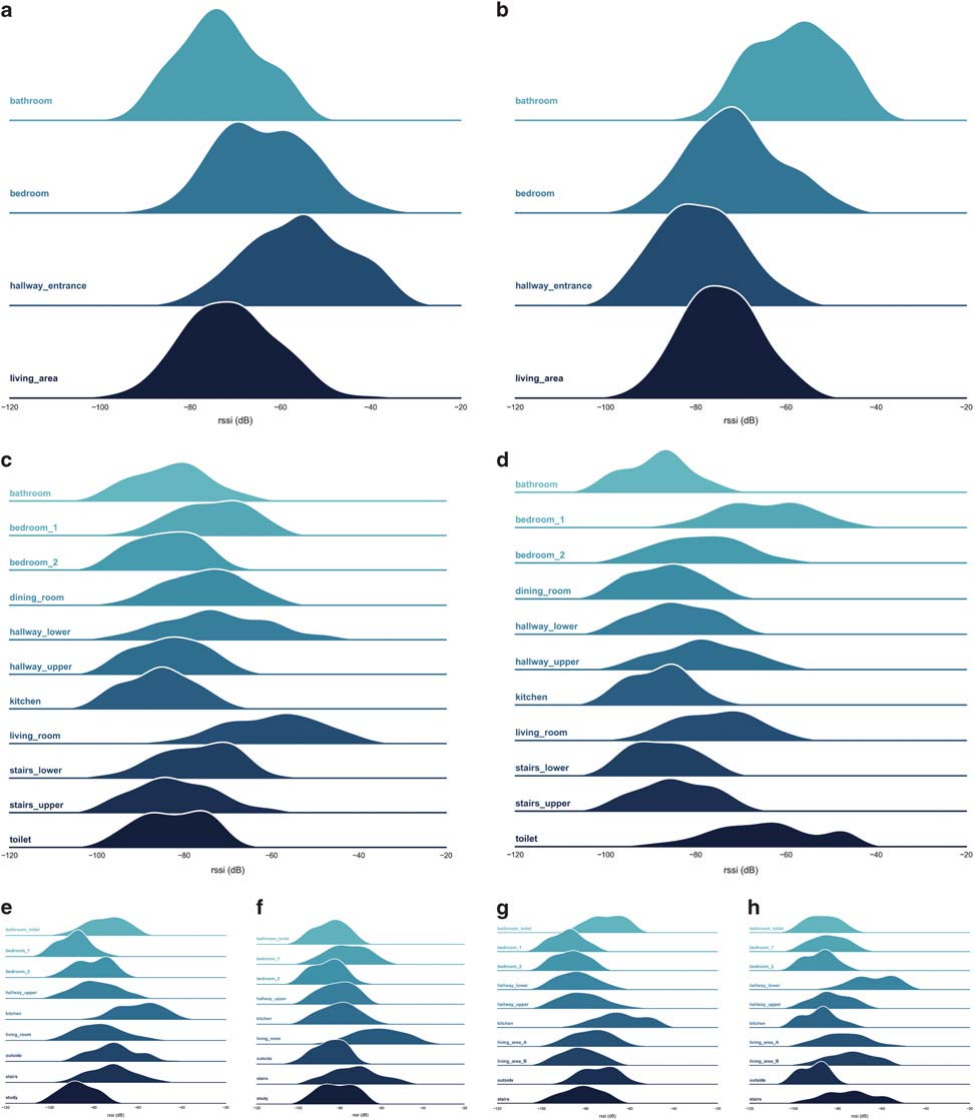
Recorded RSSI fingerprint distributions from each residence with the AP positioned in a particular room. House A with: (**a**) AP in the hallway; (**b**) AP in the bathroom. House B with: (**c**) AP in living room; (**d**) AP in bedroom 1. House C with: (**e**) AP in kitchen; (**f**) AP in living room. House D with: (**g**) AP in kitchen; (**h**) AP in the lower hallway.

The distributions in Fig. 4 illustrates Gaussian Kernel Density Estimates (KDE) of all RSS recorded by a single user when present within each room in the home. The examples show that, in most cases, the highest RSSI is registered within the room that the AP resides. In the case of 2, however, the AP located within the bedroom registers high RSSI from the toilet as well as the bedroom, where the AP was placed near the wall which adjoins both rooms (see Fig. 2c). Another example where the AP is located on the wall adjoining two rooms is highlighted in 2, with the lower hallway next to the living area B. In Fig. 4f it is evident that the AP is located in the living room and the recordings when the user was located directly above, in bedroom 1, register the highest RSS of all the rooms upstairs. This effect is also pronounced in Fig. 4g where the kitchen and the bathroom are vertically adjacent highlighting the significant signal propagation through the ceiling.

**Figure 5:**
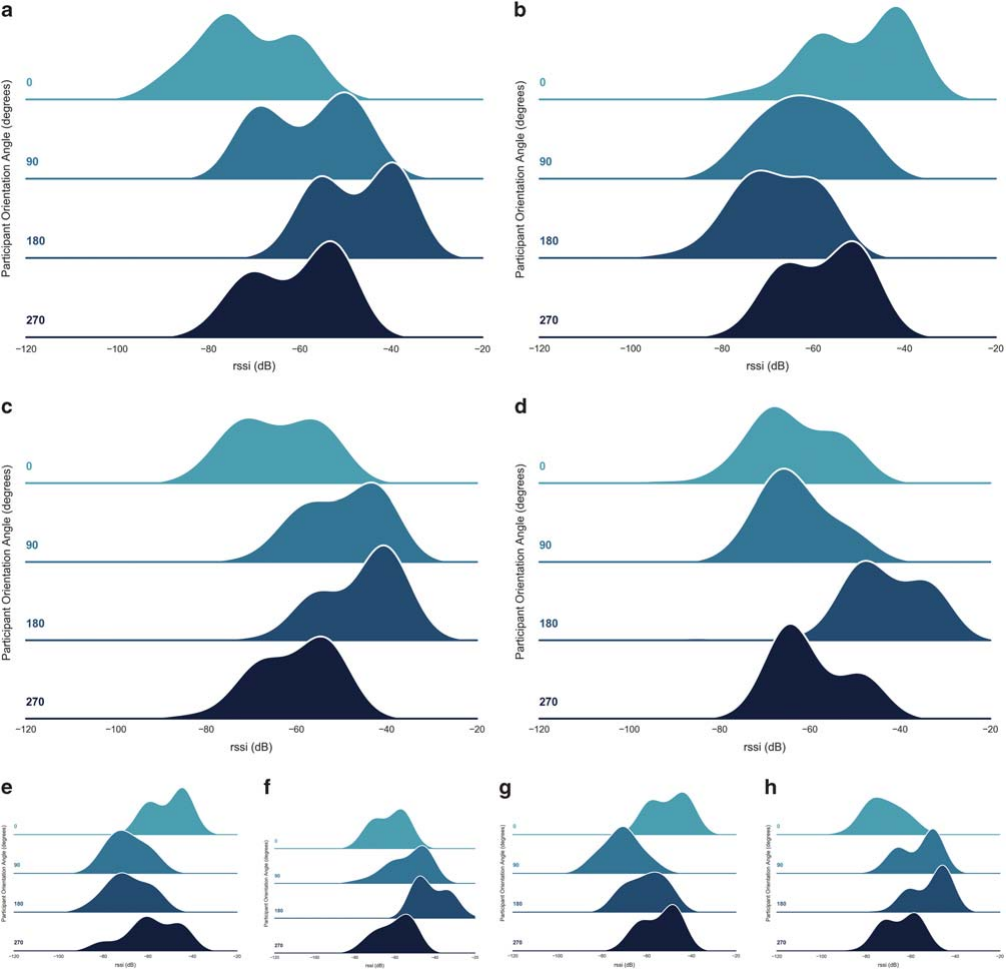
Recorded RSSI distributions of fingerprint with the user positioned at one of four orientations (relative to the tag). Distributions from house A at (**a**) tag 3, AP in bedroom, user orientated at 180° facing the AP; (**b**) tag 17, AP in living area, 0° facing AP. House B at: (**c**) tag 15, AP in kitchen, 90° facing AP; (**d**) tag 31, AP in dining room, 180° facing AP. House C at: (**e**) tag 11, AP in living room, 270° facing AP; (**f**) tag 40, AP in bedroom 1, 180° facing AP. House D at: (**e**) tag 22, AP in living area B, 0° facing AP; (**f**) tag 33, AP in bedroom 1, 180° facing AP.

Figure 5 highlight the difference in Gaussian KDE RSSI distributions captured when the participant is orientated at one of the four cardinal directions at a particular floor tag, where 0° represents floor tag north. The path between the access point and the user is unobstructed in each subplot in Fig. 5, however it is clear that the user orientation has a remarked affect on the strength of RSS recorded by the AP. At particular user orientations, the wearable is shadowed by the users body and no longer within LOS of the AP and the RSSI collected is significantly lower than the case where the wearable beam is directed, unobstructed, towards the AP. Propagation path-loss models are often used within the literature to approximate a fingerprint training scheme using simulated models. These models assume that the transmitting device (wearable) is an omnidirectional antenna source, whereas the results in Fig. 5 highlight that the beampattern of the wearable transmitter, along with the relative position of the body, significantly affects the measurements.

### Accelerometer Measurements

**Figure 6:**
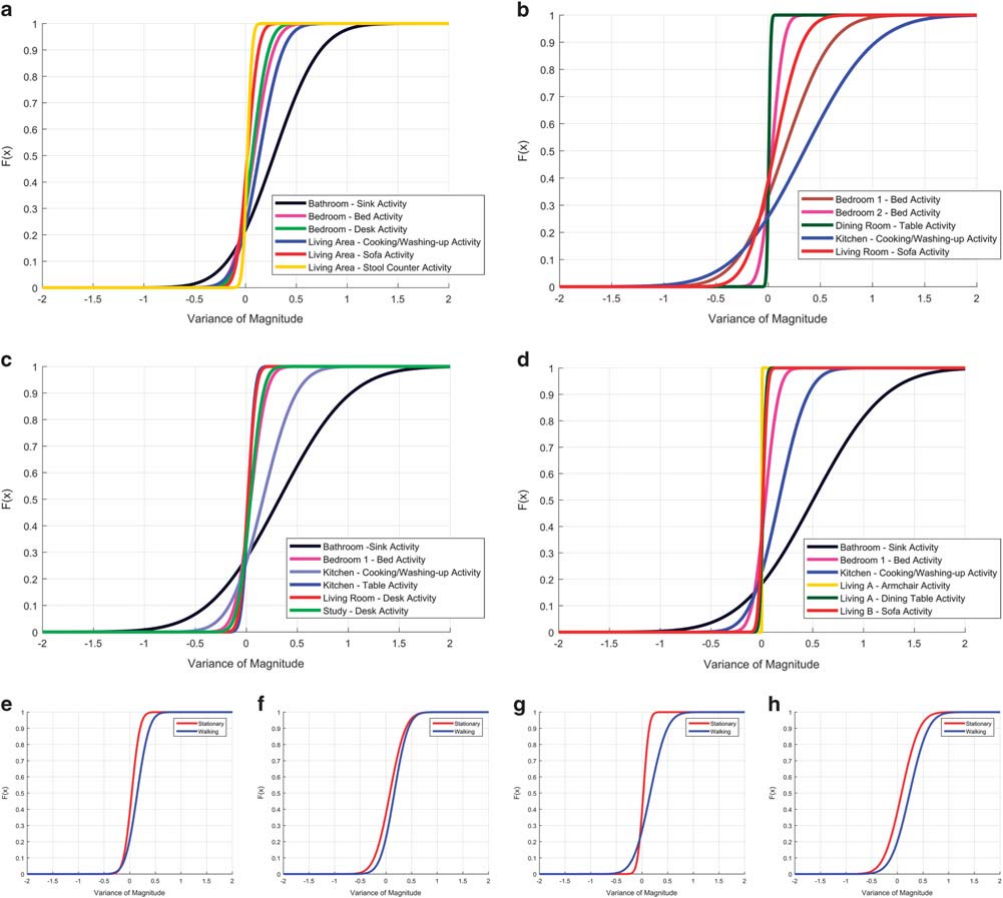
Variance of the accelerometer magnitudes in each activity zone during all the unscripted living experiments. CDFs are shown for **a**) house A, **b**) house B, **c**) house C and **d**) house D. Accelerometer variances for users when walking “W” and stationary “S” (including when standing, sitting and lying) are given in: **e**) house A; **f**) house B; **g**) house C and **h**) house D.

Figure 6 describes the Cumulative Distribution Function (CDF) of accelerometer magnitude variances recorded when the user was carrying out a selection of activities. These activities were recorded during the unscripted living experiments using the activity zone tags previously described. For each house, the living experiment accelerometer magnitude measurements were calculated and concatenated. The variance (*σ*^2^, where *σ* is the standard deviation of the series) of the accelerometer magnitude was then extracted using a sliding window of length (6.4*s*). Accelerometer measurement variance is shown for the times when the user was within an activity zone in Fig. 6a–d. The CDF was chosen to highlight the, often distinct, differences between the various activities in the same home.

While RSS measurements are region-specific and are highly dependent on the physical surroundings of the user, the accelerometer measurements for common activities are not significantly different across the four homes. The activity zone within the bathroom was recorded at the sink where the user was either washing their hands, face or teeth. These activities coincide with specific, rapid movements of the hands which register with the most significant variances in the black line plots within Fig. 6a, c and d. No bathroom activity zone measurements were present in home B (Fig. 6b). The lowest variance in each plot occurs when the participant was sitting on a stool (yellow - Fig. 6a, at the dining table (green - Fig. 6b, green - Fig. 6d), at the kitchen table (blue - Fig. 6c), at a desk (red - Fig. 6c) and in an armchair (yellow - Fig. 6d). Kitchen and bed activities register also register similar CDF curves in each of the subplots. These plots highlight how an accelerometer feature, such as variance, generalises well across the four residences, despite pertaining to different users (in the case of Fig. 6a,c and d).

Figure 6e–h describe the accelerometer magnitude variance CDF when the user was walking or standing stationary. Walking occurs less frequently than standing in the living experiments. To verify that the disparity of samples between the two labels was not detrimental, the experiments were selected, such that at least 20% of the total time was spent walking. The remainder of the extraction method was the same as above. These labels were derived by calculating velocities *d*(*s*)/*dt* from the labelled ground-truth locations using two window lengths. The smaller window (0.5s) was used in conjunction with pose information to determine when the participant was not moving. Once stationary sequences were extracted, the larger window (2s) was used on the remaining data to determine if the user was walking. Velocity and duration thresholds were used to refine the estimates. In all cases there is a difference between the red and blue line plots which represent standing and walking, respectively. The difference is, however, varied between the four homes and is least evident in the case of home B in Fig. 6f. These labels were allocated based on the camera annotations which cannot account for hand motion while the user was standing at a particular location which may influence the variance of the accelerometer magnitude.

### Video Detection

735,417 annotations were registered from the video taken during experiments in this study. On occasion, a false positive detection of tags would occur. On visual inspection of the video files, the detected tags were interpreted from small patterns on the floor or walls and typically an order of magnitude smaller than the floor tag. These were removed by thresholding the acceptable tag size detected and ensuring that subsequent detections over a short time period were not located at distances that a human could not possibly traverse. 47 detections were omitted under these constraints.

The authors recorded detailed camera calibration sequences, placing a board of tags on the floor and re-positioning themselves at various viewing angles. Despite this, when tags were detected near the periphery of the lens their relative position was often calculated incorrectly. As a result, where single tags were omitted when detected outside a specific viewing region in the frame. This filtering resulted in 94 omissions.

### Fingerprint Errors

The *fingerprint_floor* experiments were scripted. On occasion some orientations were not registered even though the user was present. In the case of tag 18 in Residence B and grid 7 in Residence A, this was due to strong sunlight falling on only a portion of the tag, inhibiting detection when the user faced particular orientations. Both fingerprints were re-annotated by hand using^20^. The only inconsistent measurement occurs at Grid 22 in house B where one out of the four orientations is not present as a result of human error.

## Usage Notes

The data repository is available from (Data Citation 1).

Codes are supplied to load the relevant data from each home and to plot the information contained in the data-descriptor. The reader is encouraged to navigate to codes/load_dataset_py/src, install the requirements and run the load_data.py example. codes/tag_detection_cpp/src contains code to perform automated tagging from a video and code to receive BLE advertising on a Raspberry Pi AP is listed in codes/ap_ble_rx_py/src.

## Additional information

**How to cite this article**: Byrne, D. *et al*. Residential wearable RSSI and accelerometer measurements with detailed location annotations. *Sci. Data* 5:180168 doi: 10.1038/sdata.2018.168 (2018).

**Publisher’s note**: Springer Nature remains neutral with regard to jurisdictional claims in published maps and institutional affiliations.

## Supplementary Material



## Figures and Tables

**Table 1:** Description of the four homes used for data acquisition.

**Home**	**Type**	**APs**	**Floors**	**Rooms**	**Duration (mins)**
A	Apartment 1 Bed	8	1	4	89
B	Terraced 2 Bed	11	2	11	171
C	Terraced 3 Bed	11	2	9	319
D	Terraced 2 Bed	11	2	10	250

**Table 2:** Description of the various activity zones and the activities pertaining to each.

**Zone**	**Activities**
Sofa	Sitting using laptop and/or watching television.
Dining Chair	Sitting eating, drinking and/or using laptop.
Desk Chair	Sitting using laptop or PC.
Bathroom Sink	Washing hands, face and/or teeth.
Kitchen Sink and Oven	Cooking and/or washing up.

**Table 3:** Description of Experiments in Residence A.

**Experiment**	**Duration (m)**	**Type**	**User**
fingerprint	32.6	Four orientations training of floor tags	a
walking_rapid	3.0	(U) walking hastily throughout the apartment	a
walking_natural	3.4	(U) walking slowly throughout	a
living_1	8.5	(U) living	d
living_2	9.2	(U) living	a
living_3	26.5	(U) living	a
living_4	5.4	(U) living	a

**Table 4:** Description of Experiments in Residence B.

**Experiment**	**Duration (M)**	**Type**	**User**
fingerprint_floor	107.0	Four orientations training of floor tags	d
fingerprint_activity	5.2	Training of activity zones	a
fingerprint_rapid	5.6	Short training of floor tags	a
walking_rapid	3.0	(U) walking hastily throughout	a
walking_natural	3.4	(U) walking slowly throughout	a
living_1	14.4	(U) living	a
living_2	7.4	(U) living	a
living_3	14.2	(U) living	d
living_4	11.2	(U) living	d

**Table 5:** Description of Experiments in Residence C.

Experiment	Duration (M)	Type	User
fingerprint_floor	71.4	Four orientations training of floor tags	c
fingerprint_activity	4.6	Training of activity zones	c
fingerprint_rapid	6.0	Short training of floor tags	c
living_1	30.7	(U) living	c
living_2	5.4	(U) living	c
living_3	22.1	(U) living	c
living_4	8.0	(U) living	c
living_5	9.7	(U) living	c
living_6	60.0	(U) living	c
living_7	30.85	(U) living	c
living_8	8.4	(U) living	c
living_9	8.6	(U) living	c
living_10	53.3	(U) living	c

**Table 6:** Description of Experiments in Residence D.

**Experiment**	**Duration (M)**	**Type**	**User**
fingerprint_floor	62.9	Four orientations training of floor tags.	d
fingerprint_activity	4.8	Training of activity zones.	d
fingerprint_rapid	4.0	Short training of floor tags.	d
living_1	29.8	(U) living	d
living_2	58.7	(U) living	d
living_3	16.9	(U) living	d
living_4	30.0	(U) living	d
living_5	43.0	(U) living	d

**Table 7:** Description of metadata files for each residence.

**File**	**Contents**
ap_mac_addresses.dat	List of APs and their MAC addresses.
ap_coordinates.dat	List of APs and their (x,y,z)m coordinates in the residence space.
ap_rooms.dat	List of APs and the rooms where they reside.
floor_tags.dat	List of floor levels in house and the floor tags plus activity zone tags present within.
room_names.dat	List of rooms in house.
room_tags.dat	List of rooms and the floor and activity zone tags present within each.
tag_coordinates.dat	List of floor tags and their coordinates.
tag_adjacency.dat	Tag numbers and their adjacent neighbouring tag.
act_desc.dat	List of activity zone tags and descriptions.
floor_<x>_tags_ap.png	Image of floor tag locations and APs where <x> is the floor.

**Table 8:** Description of files for a particular experiment from a particular house.

**File**	**Contents**
rx_wearable_data.dat	Accelerometer and RSSI recordings.
accelerometer_filtered.dat	All the recorded accelerometer data are filtered to remove redundancy presenting from simultaneous packet capture at multiple APs. Presented in x,y,z axis format.
exp_times.dat	Beginning and end timestamps of each video used for this experiment.
tag_annotations.dat	Timestamped labels of floor tags detected, relative camera coordinates and user orientation.
activity_annotation_times.dat	Timestamped manually edited annotations of activity zones given in act_desc.dat from Table 7.
wear_mac_ref.dat	Wearable MAC address for the indices described in rx_wearable_data.dat .
movement.dat	Timestamped annotations of when the user was standing still or walking. This is generated by a velocity segmentation method.
